# Genetic impacts on thermostability of onco-lncRNA HOTAIR during the development and progression of endometriosis

**DOI:** 10.1371/journal.pone.0248168

**Published:** 2021-03-05

**Authors:** Cherry Yin-Yi Chang, Chung-Chen Tseng, Ming-Tsung Lai, An-Jen Chiang, Lun-Chien Lo, Chih-Mei Chen, Man-Ju Yen, Li Sun, Li Yang, Tritium Hwang, Fuu-Jen Tsai, Jim Jinn-Chyuan Sheu

**Affiliations:** 1 Department of Obstetrics and Gynecology, China Medical University Hospital, Taichung, Taiwan; 2 School of Medicine, China Medical University, Taichung, Taiwan; 3 Institute of Biomedical Sciences, National Sun Yat-sen University, Kaohsiung, Taiwan; 4 Department of Pathology, Taichung Hospital, Ministry of Health and Welfare, Taichung, Taiwan; 5 Department of Obstetrics and Gynecology, Kaohsiung Veterans General Hospital, Kaohsiung, Taiwan; 6 School of Chinese Medicine, China Medical University, Taichung, Taiwan; 7 Human Genetic Center, China Medical University Hospital, Taichung, Taiwan; 8 Department of Gynecological Oncology, Qingdao Central Hospital, The Second Affiliated Hospital of Medical College of Qingdao University, Qingdao, People’s Republic of China; 9 Department of Gynecological Oncology, Shandong Cancer Hospital and Institute, Shandong First Medical University and Shandong Academy of Medical Sciences, Jinan, People’s Republic of China; 10 Department of Gynecology, The Third Affiliated Hospital of Zhengzhou University, Zhengzhou, China; 11 Department of Biotechnology, Kaohsiung Medical University, Kaohsiung, Taiwan; Fondazione IRCCS Ca’ Granda Ospedale Maggiore Policlinico, ITALY

## Abstract

HOTAIR is a well-known long non-coding RNA (lncRNA) involved in various cellular signaling, whereas its functional impacts on endometriosis development are still largely unknown. To this end, six potential functional single nucleotide polymorphisms (SNPs) in *HOTAIR*, with minor allele frequencies more than 10% in Han population and altered net energy of RNA structures larger than 0.5 kcal/mol, were selected for genotyping study. The study included 207 endometriosis patients and 200 healthy women. Genetic substitutions at rs1838169 and rs17720428 were frequently found in endometriosis patients, and rs1838169 showed statistical significance (*p* = 0.0174). The G-G (rs1838169-rs17720428) haplotype showed the most significant association with endometriosis (*p* < 0.0001) with enhanced HOTAIR stability, and patients who harbor such haplotype tended to show higher CA125. Data mining further revealed higher mRNA HOTAIR levels in the endometria of patients with severe endometriosis which consistently showed reduced HOXD10 and HOXA5 levels. HOTAIR knockdown with specific shRNAs down-regulated cell proliferation and migration with the induction of HOXD10 and HOXA5 expression in human ovarian clear cancer cells. Our study therefore provided evidence to indicate a prominent role of HOTAIR in promoting endometriosis, which could be used as a potential target for clinical applications.

## Introduction

Endometriosis is a clinical symptom characterized by the presence of endometrial gland and stroma outside the uterine cavity. This disease affects the life quality of around 10% of women at reproductive age. The symptoms include chronic pelvic pain, dysuria, dyspareunia, dysmenorrhea and infertility. The most common places of infringement by endometriosis are the ovaries, pelvic floor, uterine rectum concave, intestinal wall and cervix, but infringement also occurs in highly unusual places such as bronchi, lungs and brain. Therefore, endometriosis shares several characteristics of cancer such as cell migration, proliferation and anti-anoikis activity, even though endometriosis is usually considered to be a benign lesion [1,2]. Previous studies have confirmed the existence of molecular links from ovarian endometriosis (A.K.A. endometrioma) to endometrioid or clear cell-type ovarian carcinomas [1–3]. Accordingly, certain alterations in genetics or gene expression in endometriosis lesions can constitute a “perfect soil” microenvironment for malignant transformation, which deserves further investigation.

Several cancer-associated molecular alterations have also recently been detected in endometriosis. For example, driver mutations in *ARID1A*, *PIK3CA*, *KRAS*, or *PPP2R1A* can be detected in deeply infiltrating endometriosis (DIE) that virtually showed normal histological features [1,3]. In addition to genetic alterations, changes in epigenetic profiles, such as long non-coding RNAs (lncRNAs) [4,5] or miRNAs [6,7], have been reported during endometriosis development. Similarly, cancer-related functional/pathogenic single nucleotide polymorphisms (SNPs) in either miRNAs [8] or miRNA-binding sites [9] have also proven to be crucial risk factors that influence endometriosis susceptibility. The same is also true with genetic alterations in cancer-related lncRNAs, such as CDKN2B-AS and ANRIL, which have recently been reported to be associated with endometriosis development [10,11]. Those data suggest that molecular alterations for malignant transformation, either genetically or epigenetically, may serve as a driving force that triggers endometriosis development.

LncRNAs are operationally defined as non-coding RNAs with length larger than 200nt that share similar post-transcriptional features with regular mRNAs, such as RNA splicing from multi-exonic structures, transcriptional activation activity, and post-transcription 5’-capping and 3’-polyadenylation [12]. Due to diverse roles in the regulations of gene expression at both transcriptional and post-transcriptional levels, dysregulation of certain lncRNAs can contribute to the development of different human diseases including cancer [12,13]. Homeobox (HOX) transcript antisense RNA (HOTAIR) is such a unique one that has been reported to be overexpressed in a variety of human cancers [14–16]. Through binding to PRC2 by 5’-end and LSD1/CoREST/REST histone modification complexes by 3’-end, HOTAIR functions as a molecular scaffold to reprogram chromatin statuses via epigenetic silencing on target genes, such as HOX genes [16–18]. Homeoproteins function as transcription factors that can regulate expression of genes critical for embryonic development [19]. Enforced expression of HOTAIR in epithelial cancer cells increases cancer invasiveness and metastasis through reshaping epigenome, resembling embryonic fibroblasts in a manner dependent on PRC2 [20]. In ovarian cancer, emerging evidence confirms the involvement of HOTAIR in cancer progression and drug-resistance, possibly though promoting mesenchymal stem cell formation [21,22]. Using HOTAIR as a potential target for treating ovarian cancer has therefore been suggested for possible clinical practice.

Although the pro-oncogenic roles of HOTAIR in cancer have been well addressed, it remains totally unknown for its roles in the development and progression of endometriosis. In our study, we investigated genetic impacts of *HOTAIR* on endometriosis development by selecting unique functional SNPs, whose genetic variations can change the related RNA structures and thermodynamic energy. The disease-associated risk alleles/genotypes were further analyzed for their possible involvement in endometriosis pathogenesis. Gene knockdown was also performed to confirm functional relevance of HOTAIR in cell proliferation and migration of endometriosis-related ovarian cells. Findings from our study reveal a genetic linkage of HOTAIR polymorphisms to the susceptibility and pathogenesis of endometriosis.

## Methods

### Study subjects

The study population consisted of 207 patients who underwent laparotomy or laparoscopy examinations and were pathologically proven to have endometriosis at China Medical University Hospital (CMUH) in Taiwan. The guidelines from the American Society of Reproductive Medicine were applied to classify each patient’s endometriosis stage as: stage 1, minimal; stage 2, mild; stage 3, moderate; stage 4, severe [23]. Disease-related clinical information of diagnosed patients was collected from the clinical report. A total of 200 healthy women with matched age profiles were utilized as the control group. Women in this group received regular physiological checks including laparotomy or laparoscopy examinations at the same hospital, and were proven to be healthy based on examinations conducted. This study was approved by the Institutional Review Board at CMUH (CMUH105-REC1-136 and CMUH106-REC1-138) with informed consents from study subjects.

### Analyses of single nucleotide polymorphisms (SNPs) by *Taqman* genotyping

Peripheral blood leukocytes were collected from study subjects for genomic DNA extraction, using standard protocols provided by the manufacture (Genomic DNA kit; Qiagen, Valencia, CA, USA). The *Taqman* genotyping assay (Applied Biosystems Inc. Carlsbad, CA, USA) was applied to amplify DNA fragments that contain the indicated SNPs, through use of specific probes, as summarized in S1 Table.

### Thermostability predictions of HOTAIR structures influenced by the SNPs

To know the impact of each genetic substitution on local RNA secondary structure, reference and genetic-altered sequences (total 701 bp—the SNP site, plus 350 bp upstream and 350 bp downstream sequences) were submitted to RNAstructure website to generate RNA structures via the MaxExpect method (http://rna.urmc.rochester.edu/RNAstructureWeb/Servers/Predict1/Predict1.html) [24]. For the entire sequence prediction (> 2000 bp), the Mfold web server [25] was utilized to analyze HOTAIR folding (http://unafold.rna.albany.edu/?q=mfold/RNA-Folding-Form), with or without the genetic variations at the indicated SNP sites. Changes in the stability of RNA structures were compared based on free energy differences (ΔΔG). In addition, RNA samples were prepared from peripheral blood leukocytes of study subjects with different genetic variations in *HOTAIR* by using RNeasy Mini Kit (Qiagen). Quantitative RT-PCR (S2 Table) was performed to measure HOTAIR levels in those subjects using GAPDH levels as the internal controls.

### Site-directed mutagenesis

The HOTAIR cDNA clone was purchased from Addgene (#26110, Watertown, MA) and site-directed mutagenesis was performed to generate substitutions at rs1838169 and rs17720428 sites by using overlap extension PCR mutagenesis method [26]. Primers were prepared for the two-step PCR reactions (S3 Table). The old template in the PCR mixture was removed by DPN1 digestion (R0176S, NEB Inc., Ipswich, MA). The sowed PCR fragments were further digested by restriction enzymes AarI (ER1581, Thermo Fisher Scientific, Waltham, MA) and PmlI (NEB), and cloned back to the original backbone to replace the wild-type sequence. Positive clones were submitted for DNA sequencing to confirm the substitutions.

### Cell culture and HOTAIR knockdown

Clear-cell type ovarian cancer cells, ES-2 and TOV-21G, were purchased from the Bioresource Collection and Research Center (BCRC), Taiwan. Those two cells were proven to be authenticated by STR profiling (S1 and S2 Figs) and mycoplasma-free by PCR-based test (S3 Fig and S4 Table) [27]. These cells were maintained in DMEM medium, supplemented with 1% penicillin/streptomycin and 10% FBS (Gibco/Thermo Fisher Scientific). Human uterine fibroblast (HUF) cell line was purchased from ScienCell Research Laboratories (SC-7040; Carlsbad, CA) and maintained in the FM medium recommend by the company (#2301, ScienCell Research Laboratories). HOTAIR knockdown was performed by transfecting cells with a specific siRNA vector set (siRNA-A to siRNA-D) from Applied Biological Materials Inc. (#2379309; Richmond, BC). Cells treated with scramble siRNA were used as the control. The detailed target sequences can be found in S5 Table. Those vectors contain a green fluorescein protein (GFP) gene, which allowed use as a reporter to monitor gene-transfected cells. Positively transfected cells were enriched by adding G418 at a final concentration of 200 μg/ml to the culture medium 24 hrs post transfection. Cell sorting was performed for cells with low transfection efficiency (< 70% positive cells) by using a flow cytometry (Becton Dickinson, San Jose, CA). RNA samples were extracted from positively-treansfected cells by using RNeasy Mini Kit (Qiagen) and subjected to RT-PCR reaction (S2 Table) to measure mRNA levels of possible HOTAIR downstream effectors.

### Cell proliferation study

Cells transfected with siRNA constructs (B:D = 1:1 ratio) or scramble siRNA were enriched by 200 μg/ml G418 for one week followed by cell sorting. Positive cells were seeded in a 96-well plate (1×10^4^ cells/well) and cell growth was monitored for four days by MTT method. The resulting soluble formazan was developed colorimetrically at 570 nm using an ELISA reader (Molecular Devices, Sunnyvale, CA). To determine DNA synthesis activity, BrdU uptake assay was done by immuno- fluorescent staining. Briefly, cells with different constructs were seeded in a 6-well plate (5×10^5^ cells/well). After overnight, cells were pulsed with BrdU labeling solution (B23151, Thermo Fisher Scientific) for 2 hrs, followed by PBS wash and fixation with 3.7% formaldehyde in PBS. Cells were then permeabilized with 0.1% Triton X-100 and DNA was denatured with 1N HCl. After equilibrated with phosphate/citric acid buffer (182 mL of 0.2 M Na2HPO4 + 18 mL 0.1 M citric acid, pH 7.4), BrdU incorporation was detected by adding anti-BrdU monoclonal antibody/Alexa Fluor 594 (B35132, Thermo Fisher Scientific). Cell nuclei were co-stained with DAPI buffer (D1306, Thermo Fisher Scientific) and fluorescent signals (red for BrdU and blue for nuclei) were detected under an IX83 fluorescent microscope (Olympus Corp., Shinjuku, Japan).

### Cell migration study

Cells with or without HOTAIR knockdown were seeded into a 96-well plate (5×10^4^ cells/well), into which Oris stoppers (Platypus technologies, Madison, WI) was inserted. The stoppers were removed when cells had formed a confluent monolayer, and the cell migration was monitored every 12 hrs under a microscope. Cells in the detection zone were quantified by using a BioTek Synergy Multi-Mode Microplate Reader (Platypus) with an Oris detection mask attaching to the bottom of the plate.

### Statistical analysis

The allelic and genotypic frequency distributions of selected SNPs were compared between endometriosis patients and controls by chi-square analysis using the SPSS software (version 10.0, SPSS Inc. Chicago, IL, USA). The data were expressed as percentages of the total numbers of alleles and genotypes with odds ratios (ORs) and 95% confidence intervals (95% CIs), using the dominant alleles as references. Patients’ clinical features were also compared according to their genotypes or haplotypes. Independent t-test was performed to show statistical differences between the two groups. The *p*-values were adjusted, when *p*-values of f-test were larger than 0.05, according to the degree of freedom of independent t-test distributions.

## Results

### Functional single nucleotide polymorphisms (SNPs) in *HOTAIR* associate with endometriosis susceptibility

Since HOTAIR has been reported to be involved in cell proliferation and migration/invasion, we proposed that alterations in its RNA structure and function by genetic variations may contribute to endometriosis development. To prove this, a total of 20 SNP sites were filtered from the NCBI databank (https://www.ncbi.nlm.nih.gov/snp) with minor allele frequencies (MAFs) larger than 10% in the Chinese Han Beijing (CHB) population. Six potential functional SNPs were further selected whose genetic alterations can change the overall free energy of local RNA folding (ΔΔG > 0.5 or < -0.5 kcal/mol), thus possibly influencing RNA structures/functions, and which can be successfully genotyped by *Taqman* method (S1 Table; S4 Fig). Allelic and genotypic distributions indicated that people with genetic variation at either rs1838169 or rs17720428 site were at a higher risk to develop endometriosis. Significantly, the C to G alteration at rs1838169 may be a potential genetic marker that defines susceptibility to endometriosis (*p* = 0.0174; OR = 1.6852; 95% CI: 1.1924–2.3816) (Table 1). The variant G allele at rs4511324 showed a pro-disease effect, but this did not reach statistical significance. The disease-related allelic variations at SNPs rs1838169, rs4511324 and rs17720428 were recessive, such that patients with homozygous recessive genotypes were associated with higher endometriosis risk (ORs: 2.4984 for GG genotype at rs1838169, 2.9841 for GG genotype at rs4511324, and 3.9162 for GG genotype at rs17720428), even though the *p*-values were not statistically significant after Bonferronic correction (Table 2). Those data suggest genetic involvement of functional SNPs in *HOTAIR* during endometriosis development, possibly through changes in local RNA folding and stability (S5 Fig).

**Table 1:** Genotypic and allelic distributions of six functional SNPs in *HOTAIR* gene between Taiwanese endometriosis patients and controls.

SNPs^a^	Genotype/allele	No. (%) of patients	HWE^a^	No. (%) of controls	HWE^a^	*p*-value^b^	Corrected *p*-value^c^	OR (95% CI)^a^
***rs1838169***	GG	18	0.3391	7	0.7331	0.0419*	0.2514	2.8211 (1.1322–7.0293)
	GC	73		58				1.3808 (0.8928–2.1356)
	CC	103		113				1.00 Reference
	G	109 (28.09%)		67 (18.82%)		0.0029**	0.0174*	**1.6852 (1.1924–2.3816)**
	C	279 (71.91%)		289 (81.18%)				1.00 Reference
rs4511324	GG	15	0.2989	5	0.3718	0.0737	0.4422	3.1513 (1.1107–8.9406)
	GA	70		63				1.1671 (0.7646–1.7816)
	AA	119		125				1.00 Reference
	G	100 (24.51%)		73 (18.91%)		0.0560	0.1120	1.3921 (0.9906–1.9564)
	A	308 (75.49%)		313 (81.09%)				1.00 Reference
rs4512901	CC	15	0.2989	6	0.6116	0.1232	0.7392	2.6261 (0.9861–6.9934)
	CA	70		62				1.1860 (0.7760–1.8124)
	AA	119		125				1.00 Reference
	C	100 (24.51%)		74 (19.17%)		0.0693	0.4158	1.3689 (0.9750–1.9219)
	A	308 (75.49%)		312 (80.83%)				1.00 Reference
rs4759313	TT	26	0.365	14	0.7655	0.1421	0.8526	2.0186 (0.9937–4.1008)
	TA	85		79				1.1695 (0.7706–1.7748)
	AA	92		100				1.00 Reference
	T	137 (33.74%)		107 (27.72%)		0.0664	0.3984	1.328 (0.9805–1.7985)
	A	269 (66.26%)		279 (72.28%)				1.00 Reference
rs10783616	CC	25	0.4608	14	0.7864	0.1557	0.9342	1.9819 (0.9715–4.0432)
	CG	85		79				1.1942 (0.7868–1.8125)
	GG	91		101				1.00 Reference
	C	135 (33.58%)		107 (27.58%)		0.0672	0.4032	1.3278 (0.9798–1.7996)
	G	267 (66.42%)		281 (72.42%)				1.00 Reference
***rs17720428***	GG	16	0.2359	4	0.2141	0.0286*	0.1716	**4.1333 (1.3432–12.7195)**
	GT	71		63				0.9169 (0.6012–1.3983)
	TT	120		124				1.00 Reference
	G	103 (24.88%)		71 (18.59%)		0.0318*	0.1908	**1.4507 (1.0318–2.0397)**
	T	311 (75.12%)		311 (81.41%)				1.00 Reference

^a^Abbreviations: SNP, single-nucleotide polymorphism; HWE, Hardy-Weinberg equilibrium testing; OR, odds ration; 95% CI, 95% confidence interval.

^b^*p*-values were calculated by χ2 test.

^c^Bonferroni correction was applied to get the corrected *p*-value, which equals to *p*-value×6. Statistical significance

*, *p*-value <0.05

**, *p*-value <0.01

***, *p*-value <0.001.

**Table 2:** Dominant and recessive effects of the selected SNPs in *HOTAIR* gene between Taiwanese endometriosis patients and controls.

SNPs^a^	Genotype	No. (%) of patients	No. (%) of controls	*p*-value^b^	Corrected *p*-value^c^	OR (95% CI)^a^
***rs1838169***	GC+GG	91	65	0.0424*	0.2544	**1.5359 (1.0138–2.3271)**
	CC	103	113			1.00 Reference (dominant)
	GG	18	7	0.0397*	0.2382	**2.4984 (1.0177–6.1331)**
	CC+GC	176	171			1.00 Reference (recessive)
***rs4511324***	GA+GG	85	68	0.1884	1.0000	1.3130 (0.8750–1.9703)
	AA	119	125			1.00 Reference
	GG	15	5	0.0302*	0.1812	**2.9841 (1.0631–8.3761)**
	AA+GA	189	188			1.00 Reference
rs4512901	CA+CC	85	68	0.1884	1.0000	1.3130 (0.8750–1.9703)
	AA	119	125			1.00 Reference
	CC	15	6	0.0754	0.4524	2.3545 (0.8938–6.2026)
	AA+CA	189	187			1.00 Reference
rs4759313	TA+TT	111	93	0.1963	1.0000	1.2973 (0.8739–1.9259)
	AA	92	100			1.00 Reference
	TT	26	14	0.0668	0.4008	1.8781 (0.9495–3.7151)
	AA+TA	177	179			1.00 Reference
rs10783616	CG+CC	110	93	0.1773	1.0000	1.3128 (0.8839–1.9498)
	GG	91	101			1.00 Reference
	CC	25	14	0.0822	0.4935	1.8263 (0.9193–3.6283)
	GG+CG	176	180			1.00 Reference
***rs17720428***	GG+GT	87	67	0.1552	0.9314	1.3418 (0.8944–2.0129)
	TT	120	124			1.00 Reference
	GG	16	4	0.0101*	0.0608	3.9162 (1.2854–11.9317)
	TT+GT	191	187			1.00 Reference

^a^Abbreviations: SNP, single-nucleotide polymorphism; OR, odds ration; 95% CI, 95% confidence interval.

^b^*p*-values were calculated by χ2 test.

^c^Bonferroni correction was applied to get the corrected *p*-value, which equals to *p*-value×6. Statistical significance

*, *p*-value <0.05

**, *p*-value <0.01

***, *p*-value <0.001.

### Haplotype analyses of functional SNPs in *HOTAIR* and associations with clinical features

**Fig 1:**
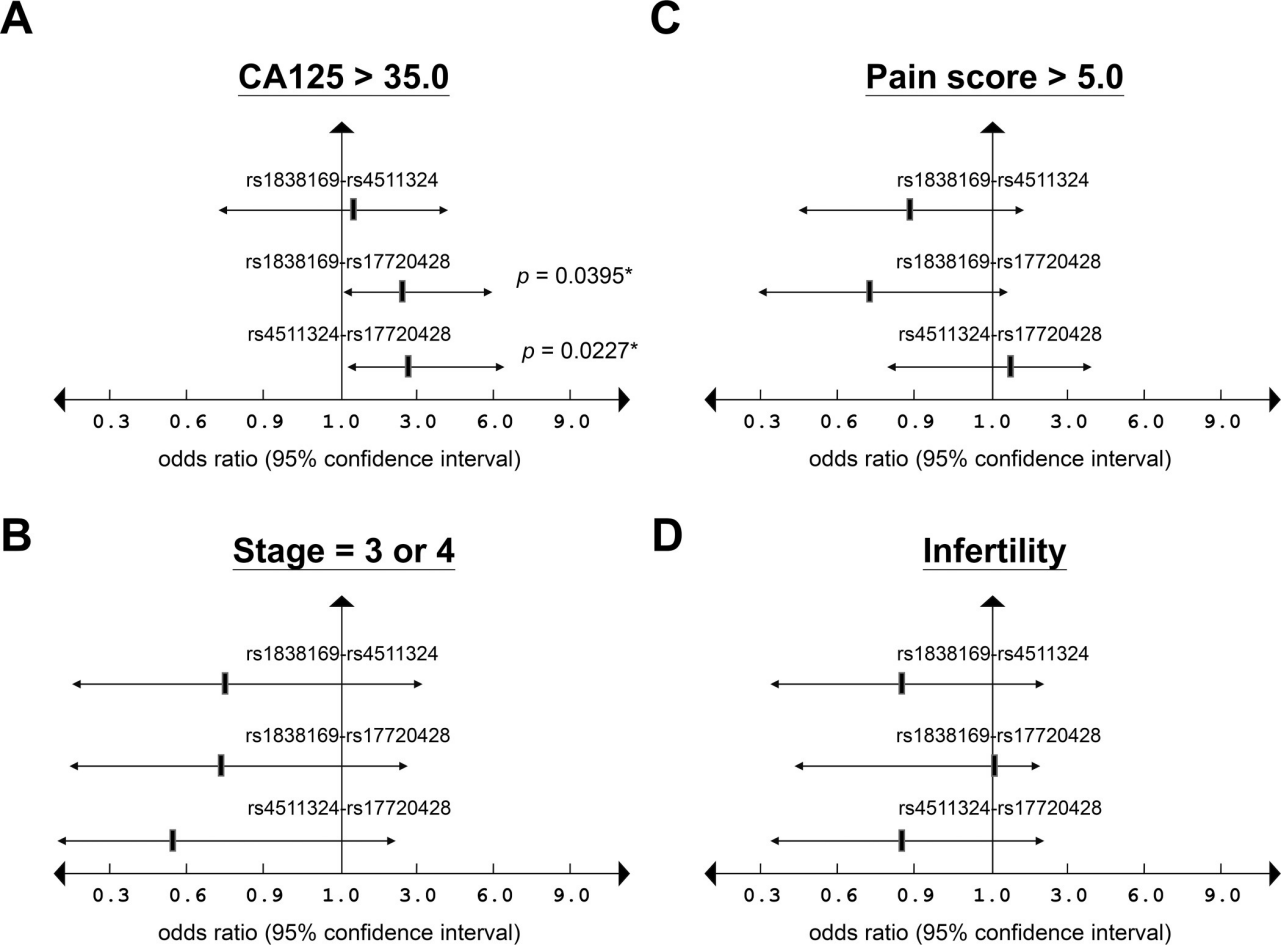
Haplotypes of endometriosis-related SNPs in *HOTAIR* and their associations with clinical features. Two-allele genetic combinations among three endometriosis-related SNPs in *HOTAIR*, including rs1838169, rs4511324 and rs17720428, were analyzed in patients and correlated with the frequencies of (A) high CA125 levels (> 35.0 U/ml); (B) severe pain (pain score > 5.0); (C) advanced stages (stages 3 and 4); and (D) infertility.

Haplotype frequencies were further analyzed for those three disease-related functional SNPs to ascertain their combinational impacts on endometriosis pathogenesis. Consistent with allelic distribution data, patients with haplotypes of two pro-disease alleles were at a high risk to develop endometriosis, whereas haplotypes with two reference alleles represented protective effects against the disease (Table 3). Among two-allele combinations, patients with the G-G haplotype of rs1838169-rs17720428 showed the most significant association with endometriosis development (*p* = 0.0003; OR = 1.8463; 95% CI: 1.3512–2.5228). By contrast, patients with the C-T haplotype of rs1838169-rs17720428 showed the lowest susceptibility to this disease (*p* = 0.0002; OR = 0.6250; 95% CI: 0.5004–0.7806). When compared with clinical features, the G-G haplotype of rs1838169-rs17720428 and the G-G haplotype of rs4511324-rs17720428 were found to be associated with higher serum CA125 levels (> 35.0 U/ml) (*p*-values were 0.0395 and 0.0227, respectively) (Fig 1). Our data therefore confirmed potent roles of *HOTAIR* functional SNPs in endometriosis development, especially for the genetic combination between allelic variations at rs1838169 and rs17720428.

**Table 3:** Haplotype frequencies of selected SNPs in *HOTAIR* gene between endometriosis patients and controls.

SNPs^a^	Genotype/allele	No. (%) of patients	No. (%) of controls	*p-*value^b^	Corrected *p*-value^c^	OR (95% CI)^a^
rs1838169	GG	130	73	0.0004***	0.0024**	**1.7342 (1.2760–2.3571)**
rs4511324	CG	64	53	0.6171	1.0000	1.1013 (0.7538–1.6090)
	GA	88	66	0.2222	1.0000	1.2331 (1.2331–1.7269)
	CA	498	514	0.0002***	0.0012**	**0.6597 (0.5289–0.8228)**
rs1838169	GG	129	69	<0.0001***	0.0003***	**1.8463 (1.3512–2.5229)**
17720428	GT	85	63	0.1884	1.0000	1.2589 (0.8932–1.7742)
	CG	69	55	0.4348	1.0000	1.1582 (0.8000–1.6768)
	CT	489	517	<0.0001***	0.0002***	**0.6250 (0.5004–0.7806)**
rs4511324	GG	130	73	0.0002***	0.0012**	**1.7939 (1.3215–2.4351)**
17720428	GT	70	63	0.8065	1.0000	1.0437 (0.7313–1.4896)
	AG	74	63	0.5657	1.0000	1.1093 (0.7805–1.5767)
	AT	538	561	0.0011**	0.0066**	0.9355 (0.7445–1.1754)

^a^Abbreviations: SNP, single-nucleotide polymorphism; OR, odds ration; 95% CI, 95% confidence interval.

^b^*p*-values were calculated by χ2 test.

^c^Bonferroni correction was applied to get the corrected *p*-value, which equals to *p*-value×6. Statistical significance

*, *p*-value <0.05

**, *p*-value <0.01

***, *p*-value <0.001.

### Alterations in HOTAIR structures and functional impacts on endometriosis development

**Fig 2:**
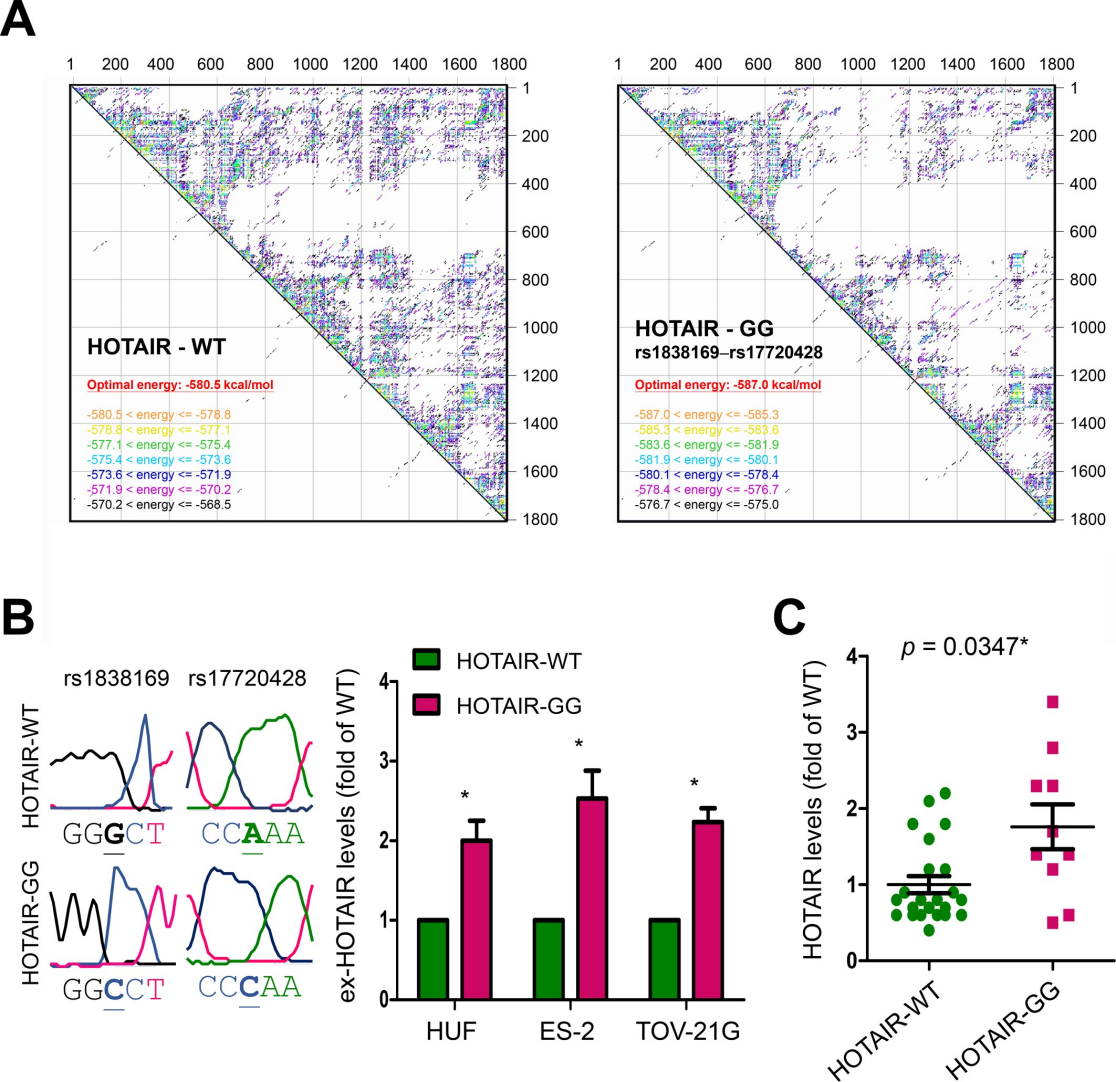
Thermo-stability of HOTAIR effected by genetic variations. (A) Thermo-stability of full-length HOTAIRs with the risk G-G haplotype of rs1838169-rs17720428 (HOTAIR—GG) or wide type (HOTAIR—WT) were estimated by RNA-folding algorithm from the Mfold web server (http://unafold.rna.albany.edu/?q=mfold/RNA-Folding-Form). (B) HOTAIR-WT or HOTAIR-GG vector (G-G haplotype of rs1838169-rs17720428) were prepared (*left*). Please note, HOTAIR is transcribed by the negative strand. Quantitative RT-PCR was performed (n = 3) to measure the exogenous HOTAIR (ex-HOTAIR) levels in cells transfected with different constructs (*right*). The data were normalized using the levels of puromycin resistant gene expressed by the vectors (S2 Table). (C) Quantitative RT-PCR was performed to measure relative HOTAIR levels in peripheral blood leukocytes of study subjects with the risk G-G haplotype of rs1838169-rs17720428 (HOTAIR-GG; n = 10) and subjects with wild type genetic background (HOTAIR-WT; n = 23). For data in (B) and (C), *t*-test was applied to compare the differences between HOTAIR-WT and HOTAIR-GG groups. The *p* values were presented as *: *p* value < 0.05, **: *p* value < 0.01, and ***: *p* value < 0.001.

To understand why allelic variants at rs1838169 and rs17720428 could lead to endometriosis susceptibility, we analyzed the changes in thermos-stability of HOTAIR structure caused by genetic substitutions at both rs1838169 and rs17720428, the G-G haplotype. As shown in Fig 2A, the optimal net energy for wild type HOTAIR was estimated as -580.8 kcal/mol, with more than 62% inter-residue interactions showing net energy larger than -580.8 kcal/mol. For the HOTAIR variant containing nucleotide substitutions at rs1838169 and rs17720428, the optimal net energy was estimated as -587.0 kcal/mol, with less than 23% inter-residue interactions showing net energy higher than -580.8 kcal/mol. Wild type HOTAIR (HOTAIR-WT) and its variant with G-G haplotype of rs1838169-rs17720428 (HOTAIR-GG) were prepared and transfected into human uterine fibroblast (HUF), and two ovarian cancer cell lines, ES-2 and TOV-21G cells. Two days after the transfection, the exogenous HOTAIR (ex-HOTAIR) levels in treated cells were analyzed by quantitative RT-PCR (S2 Table). Our data confirmed higher ex-HOTAIR levels in HOTAIR-WT transfected cells than in HOTAIR-WT treated cells (Fig 2B). This finding can be further supported by analyzing HOTAIR levels in peripheral blood leukocytes of study subjects with different genetic statuses. As shown in Fig 2C, HOTAIR levels in blood cells from subjects with the risk G-G haplotype of rs1838169-rs17720428 are higher (about 1.8 fold) than that in subjects with WT genetic background (*p* = 0.0347). The above findings indicated a more stable HOTAIR structure for the variant containing the two major functional SNPs of rs1838169 and rs17720428, suggesting elevated HOTAIR levels in persons with endometriosis susceptibility.

**Fig 3:**
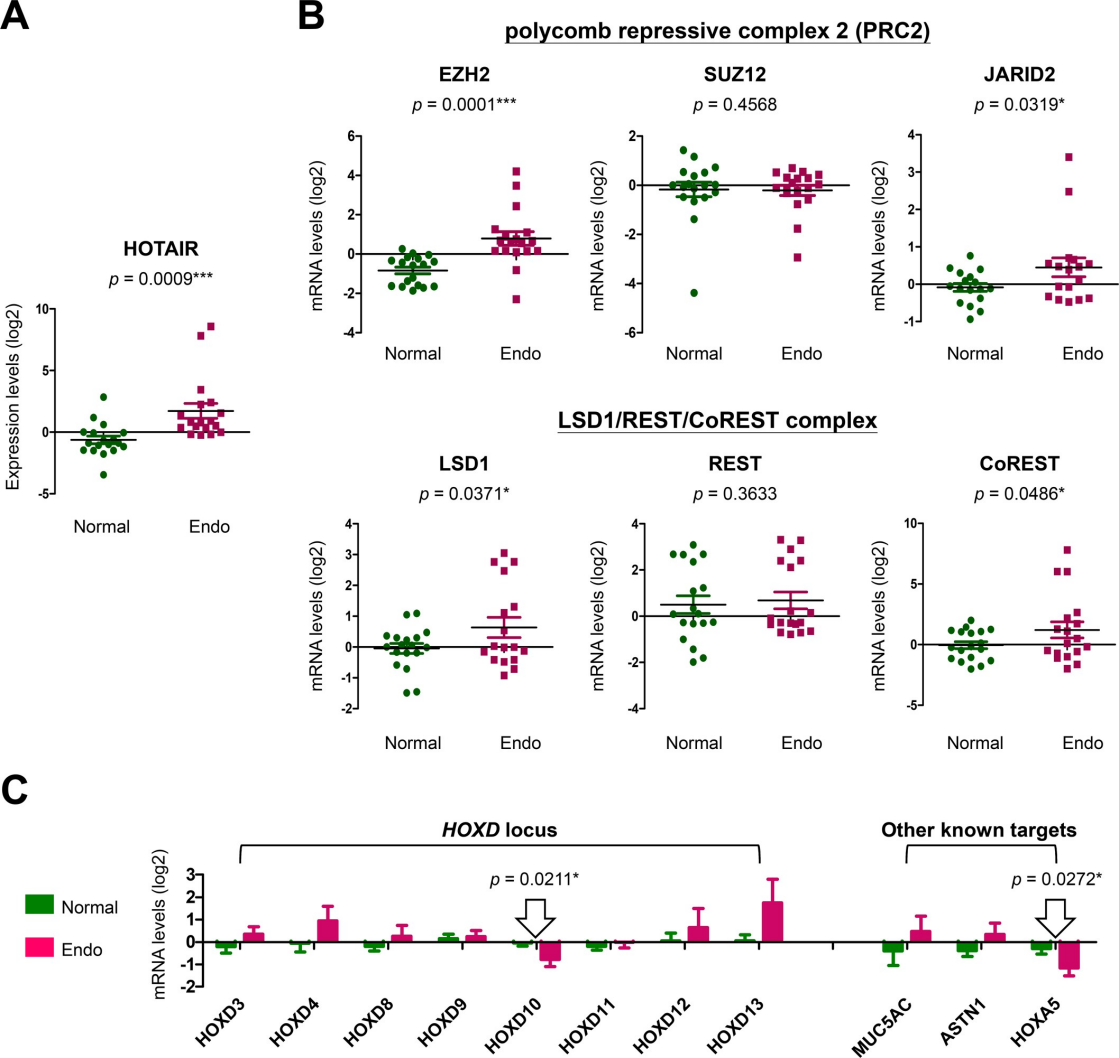
HOTAIR expression and its association with endometriosis development. Microarray data (GSE120103) from the GEO databank was utilized for clinical association study. (A) HOTAIR levels in lesions from severe endometriosis patients (stage 4) (Endo; n = 18) were analyzed and compared with that in normal endometria (Normal; n = 18). (B) Expression levels of component genes in HOTAIR-associated complexes, PRC2 and LSD1/REST/CoREST, were also analyzed and compared with the levels in healthy controls. (C) Expression levels of possible downstream effectors of HOTAIR, including genes at the *HOXD* locus and other validated targets [17,28], were compared between patients (Endo) and controls (Normal) by using *t*-test.

To prove this, microarray data (GSE120103) from the GEO databank was utilized to analyze gene expression levels. We found that patients with aggressive endometriosis (at stage 4) expressed higher HOTAIR levels in their endometria as compared to normal controls without endometriosis (*p* = 0.0009) (Fig 3A). Notably, we also found a functional relevance of the elevated HOTAIR levels, showing that key components of HOTAIR-associated epigenetic regulators were also upregulated in endometriosis patients, including EZH2 and JARID2 (to form histone methyltransferase PRC2 complex), as well as LSD1 and CoREST (to form histone demethylase LSD1/REST/CoREST complex) (Fig 3B). The most responsive downstream targets, genes at the *HOXA* and *HOXD* loci [17,18], that associated with elevated HOTAIR in severe endometriosis were HOXD10 and HOXA5 (Fig 3C). Our results support a potent role for elevated HOTAIR in endometriosis development.

### Anti-HOTAIR suppresses cell proliferation and mobility of ovarian clear cancer cells

**Fig 4:**
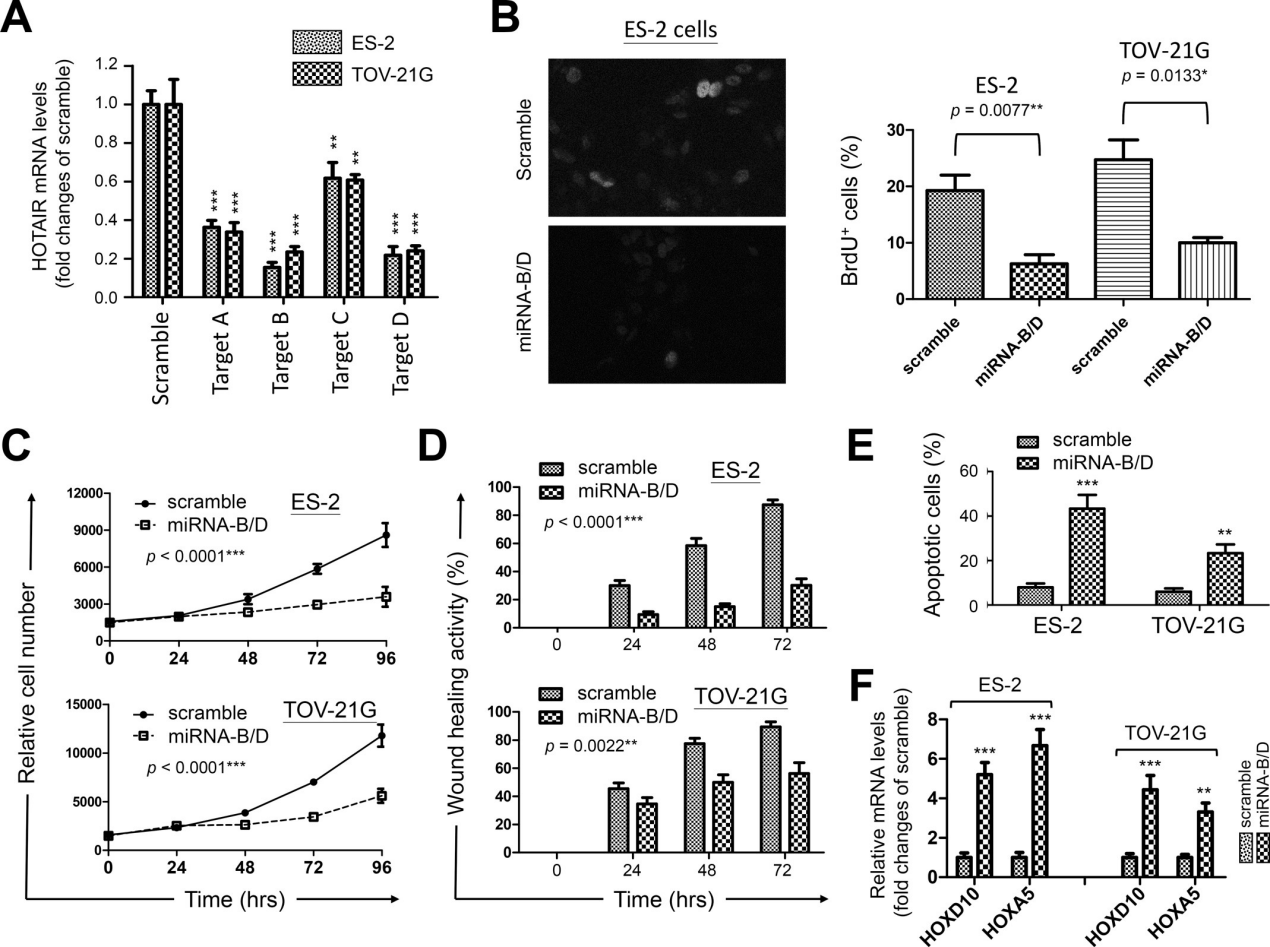
Functional impacts of HOTAIR knockdown on survival activity of ovarian clear cancer cells. (A) Specific miRNAs (set A to D) against HOTAIR were prepared and introduced into ovarian clear cancer cells, ES-2 and TOV-21G. Gene knockdown efficiency of each miRNA construct was verified by QPCR using scramble miRNA as the control. Data were averages of triplicates and presented as means ± S.D. (B) BrdU uptake assay was performed in cells transfected with scramble control or the mixture of anti-HOTAIR miRNA-B and miRNA-D (miRNA-B/D) (*left*). Positive cells were counted and averaged as means ± S.D. from 200 nuclei (*right*). (C) MTT assay and (D) wound healing assay were performed to investigate the impacts of HOTAIR knockdown by miRNA-B/D on cell growth and migration, respectively. (E) Apoptotic cells were detected by annexin V staining in cells 72 hrs after transfection. Data in (C) and (D) were averages of five replicates and presented as means ± S.D. (F) The expression levels of HOXD10 and HOXA5 were analyzed by QPCR in transfected cells 24 hrs after HOTAIR knockdown. Cells treated with scramble were utilized as the controls. Data were averages of triplicates and presented as means ± S.D. For (A), (B), (E) and (F), data from miRNA-B/D treated cells were compared to the ones from scramble control by using *t*-test. For (C) and (D) time course studies, two-way ANOVA was utilized to analyze statistical significance. The *p* values were presented as *: *p* value < 0.05, **: *p* value < 0.01, and ***: *p* value < 0.001.

Since HOTAIR has been considered as an onco-lncRNA [14,15,20], we next asked the possibility of using HOTAIR as a potential target for treating endometriosis and the associated malignancies. Two ovarian cancer cell lines, ES-2 and TOV-21G which were believed to arise in endometriosis background [29], were utilized in a gene knockdown study. A set of anti-HOTAIR miRNAs were tested (S5 Table) and miRNAs targeting regions B and D provided us with the best knockdown efficiency (Fig 4A). When introduced into ovarian clear cancer cells, anti-HOTAIR miRNA-B/D significantly suppressed nucleotide uptake, cell growth and mobility, whereas the scramble miRNA control showed limited effects (Fig 4B–4D). Cell viability assay by annexin-V staining revealed increased apoptotic cells triggered by HOTAIR knockdown (Fig 4E). To confirm the biological impact, mRNA levels of HOXD10 and HOXA5 were found elevated after HOTAIR down-regulation by QPCR (Fig 4F). Similar experiments were also performed on HUF cells as a normal control. Limited effects on DNA replication and cell growth were found in HUF cells after HOTAIR knockdown, possibly due to a relatively lower HOTAIR level in HUF cells than in cancer cells (S6A–S6D Fig). Slight reduction of cell migration was observed which correlated with an increase of HOXD10 and HOXA5 levels (S6E Fig). Our data confirm the molecular link between HOTAIR and those homeobox proteins, suggesting the involvement of HOXD10 and HOXA5 in endometriosis progression.

## Discussion

In this study, we discovered that genetic variations in *HOTAIR*, especially the SNPs at rs1838169-G and rs17720428-G alleles, can change local RNA structures (S1 Table and S5 Fig) and define the susceptibility to endometriosis (Tables 1 and 2). People with the genetic combination of risk alleles at these two functional SNPs (haplotype G-G) are at a high risk to develop endometriosis (Table 3) with higher serum CA125 levels (Fig 1). Molecular modeling confirmed HOTAIR stabilization via genetic substitutions at both SNPs through reduction of the free-energy for RNA folding, resulting in the enhancement of HOTAIR function (Fig 2). Data mining of transcriptome profiles in endometrial tissues also indicated higher HOTAIR levels and functions in endometriosis patients at more advanced stages, suggesting potent roles in disease progression (Fig 3). Gene knockdown by specific miRNAs reduced cell growth and migration, leading to increased cell death in endometriosis-associated malignant ES-2 and TOV-21G cells (Fig 4). Downstream screening using data from clinical and cell-based studies revealed HOXD10 and HOXA5 to be the key regulators for HOTAIR to promote endometriosis progression (Figs 3C and 4F). To our knowledge, this is the first report to address the potent roles played by functional SNPs in *HOTAIR* during the development and progression of endometriosis.

**Fig 5:**
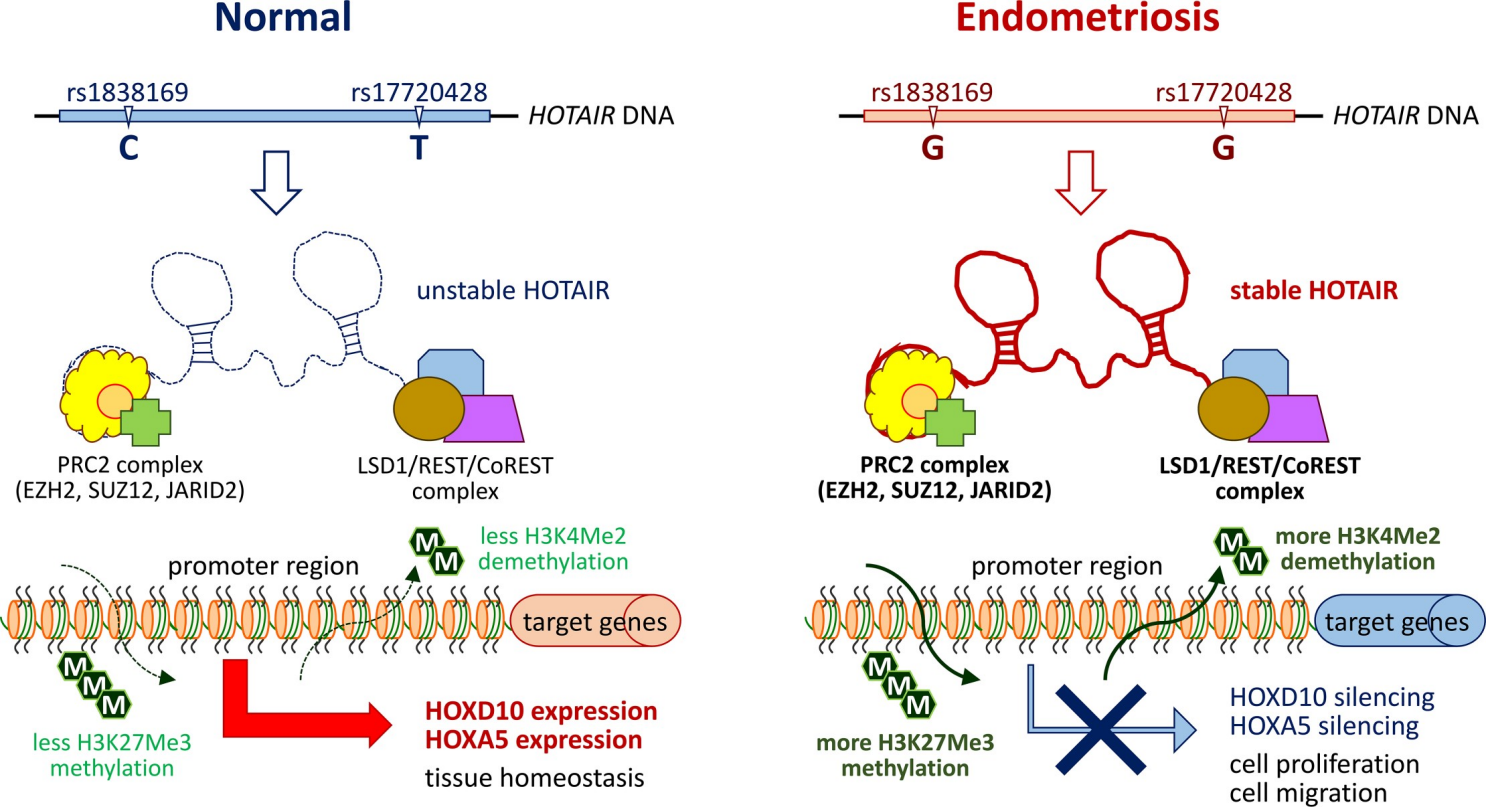
Schematic summary of a proposed model depicting functional impacts of *HOTAIR* genetic variations on endometriosis development. Genetic variations at SNP sites, rs1838169 (C to G) and rs17720428 (T to G), in *HOTAIR* are frequently detected in patients with endometriosis. Such genetic substitutions alter thermo-stability of mature HOTAIR and stabilize its RNA structure. Through epigenetic silencing by H3K27 tri‐methylation or H3K4-demethylation, higher HOTAIR levels in endometriosis patients can down-regulate its down-stream effectors, such as HOXD10 and HOXA5, leading to increased cell proliferation and migration/invasion.

HOTAIR is well-known for its functional roles in regulating the expression of different homeoproteins (HOXs) with tissue and cell type-dependent effects. Controlling proper dynamics of HOX network is critical to maintaining endometrium homoeostasis during embryonic implantation or menstrual cycles [30]. For example, alternation of HOXA10 and HOXA11 expression has been identified as a mechanism of infertility associated with endometriosis [30,31]. Molecularly, the 5’-end of HOTAIR interacts with and guides PRC2 complex, a histone methylase, to the *HOXD* locus, thus silencing the subsequent transcription via histone H3 lysine 27 (H3K27) tri‐methylation on target genes [17,18,32]. On the other hand, the 3’-end of HOTAIR was also found to interact and recruit LSD1 (A.K.A. KDM1)/REST/CoREST complex, a histone demethylase, to the target loci, resulting in target gene silencing via H3K4-demethylation [17,18]. Recent study has provided evidence to suggest HOXA5 as one of HOTAIR responsive downstream effectors [31]. Our study identified two major downstream targets, HOXD10 and HOXA5, regulated by HOTAIR in advanced endometriosis and the associated ovarian clear cancer cells. Although our data did not show the linkage with infertility, previous study did find that these two proteins play negative regulatory roles in cell proliferation, migration/invasion and angiogenesis [33–35]. Down-regulation of these two targets by HOTAIR stabilization and elevation at advanced endometriosis may provide an advantageous microenvironment for lesion growth and spread to other organs (Fig 5). Whether such a regulatory axis serves as a driving force for the associated malignant transition requires further study.

The involvement of *HOTAIR* functional SNPs in oncogenesis has been previously reported in a variety of cancer types, including ovarian cancer [36,37]. In addition to the association of disease susceptibility, several studies also indicated the clinical relevance of those genetic variations to cancer progression. The most frequently studied functional SNPs include genetic variations at the intron enhancer region, such as rs4759314-G allele and rs920778-T allele, which showed transcription-promoting activity, leading to increased total HOTAIR levels [38,39]. Another intron variant, rs1899663-A allele, had a significantly increased cancer risk [40], whereas the SNP within the exon region, rs7958904-C allele, was found to play a protective role against cancer [41]. To date, the detail mechanisms associated with such substitutions in cancer development are still unclear. More sophisticated strategies should be considered to screen for other disease-related functional SNPs whose genetic substitutions can have direct impacts on HOTAIR structure, expression or activity.

In addition to ovarian cancer, HOTAIR upregulation was also observed in other gynecological cancers such as cervical and endometrial carcinoma, and its expression levels correlated with poor clinical outcomes [42]. Our study also indicated that HOTAIR levels determined the sensitivity of cell growth arrest and cell death triggered by HOTAIR knockdown (Figs 4 and S6). On the other hand, recent study confirmed the miR-646/NPM1 axis, an indicator for total active nucleoli, as critical for HOTAIR to control estrogen-induced viability, migration, and invasion of endometrial carcinoma cells [43]. This finding may support the point of view that enhanced proliferative capacity by facilitating ribosome biogenesis may be one of the mechanisms driven by HOTAIR. Interestingly, we have previously demonstrated that upregulation of ribosome biogenesis including NPM1 by the oncogenic mir196a2 variant was one of key mechanisms involved in the malignant transition from endometriosis to the related ovarian cancer [8]. Clinical observations in cancer therapy have provided convincing evidence to suggest the hyperactive nucleolus as an emerging target for chemotherapeutic intervention [44,45]. Since oncogenic lncRNAs like HOTAIR can interact with many miRNAs as decoys or sponges to establish competing endogenous RNA (ceRNA) networks for malignant transformation [12,16], whether HOTAIR forms a complex with mir196a2 to promote endometriosis progression is worthy of further investigation. Furthermore, a combination of anti-nucleolus drugs with anti-HOTAIR might be beneficial for patients with advanced endometriosis and the associated malignancies.

Due to limited sample size in this study, we only focused on SNPs with an MAF >10% to minimize possible biases arising from small sample numbers. To consider functional relevance, we used sufficient energy changes (ΔΔG > 0.5 or < -0.5 kcal/mol) as another filter to pick up novel SNPs in *HOTAIR*. Through this approach, we selected six potent SNPs, including two in the upstream transcriptional region (rs4511324 and rs4512901), two in the intron enhancer region (rs4759313 and rs10783616), and two in the exon region (rs1838169 and rs17720428). SNPs rs4759314, rs920778 and rs7958904 were previously reported to be risk genetic factors for ovarian cancer [36,37], however, they were not included in this study because the MAF of rs4759314 in the Chinese Han population is less than 10% and genetic substitutions at rs920778 and rs7958904 caused no local energy change (S1 Table). Based on our data, we discovered that genetic variations in the exon region (rs1838169 and rs17720428) can potentially stabilize HOTAIR structure (ΔΔG = -6.5 kcal/mol) and showed an increased risk for endometriosis development. Whether these two functional SNPs define a genetic predisposition to the associated cancers and participate in cancer development requires a more well-defined study.

Some limitations of this study need to be addressed here. Firstly, the functional SNPs (rs1838169 and rs17720428) defined in this study were not included in previously reported GWAS studies on cohorts with Asian ethnicity [46–48]. Study using another cohort is required to further confirm genetic susceptibility of these two SNPs to endometriosis. In addition, functional validation of HOTAIR/HOXD10 and HOTAIR/HOXA5 axes should be further confirmed in benign endometriotic cells to see their impacts on tumorigenesis, which will be investigated in our future study.

## Conclusions

We demonstrated in this study that genetic alterations in *HOTAIR* may be one of the risk factors leading to endometriosis development (Fig 5). Increased HOTAIR levels arising from thermo-stabilization could be one of possible mechanisms underlying the genetic susceptibility. In addition, our data indicated that functional axes of HOTAIR/HOXD10 and HOTAIR/HOXA5 may play potent roles in regulating endometriosis progression. Targeting HOTAIR is a potential strategy for treating endometriosis and suppressing further malignant transition.

## Supporting information

S1 Fig DNA typing report of ES-2 cells utilized in this study. (PDF)

S2 Fig DNA typing report of TOV-21G cells utilized in this study. (PDF)

S3 Fig PCR test for detecting mycoplasma contamination. (PDF)

S4 Fig Allelic discrimination plots of the six functional single-nucleotide polymorphisms (SNPs) in *HOTAIR* gene. (PDF)

S5 Fig Functional impacts of endometriosis-related risk alleles (SNPs at rs1838169, rs4511324 and rs17720428) on HOTAIR RNA structure. (PDF)

S6 Fig Functional impacts of HOTAIR knockdown on survival activity of human uterine fibroblast (HUF) cells. (PDF)

S1 Table Summary of the functional SNPs in *HOTAIR*. (PDF)

S2 Table Primer sequences and amplification program for quantitative RT-PCR reactions in this study. (PDF)

S3 Table Primer sequences and PCR amplification program for site-directed mutagenesis in this study. (PDF)

S4 Table Primer sequences and amplification program for PCR-based mycoplasma test. (PDF)

S5 Table Target sequences of siRNA vectors used in this study. (PDF)
