# Supplementary material for: Genetic impacts on thermostability of onco-lncRNA HOTAIR during the development and progression of endometriosis
Source: PLoS One. 2021 Mar 5;16(3):e0248168. doi: 10.1371/journal.pone.0248168 (PMC7935326; doi:10.1371/journal.pone.0248168)
Supplement: S2 Fig — (PDF) [file pone.0248168.s002.pdf]

STR profiling of TOV-21G cells

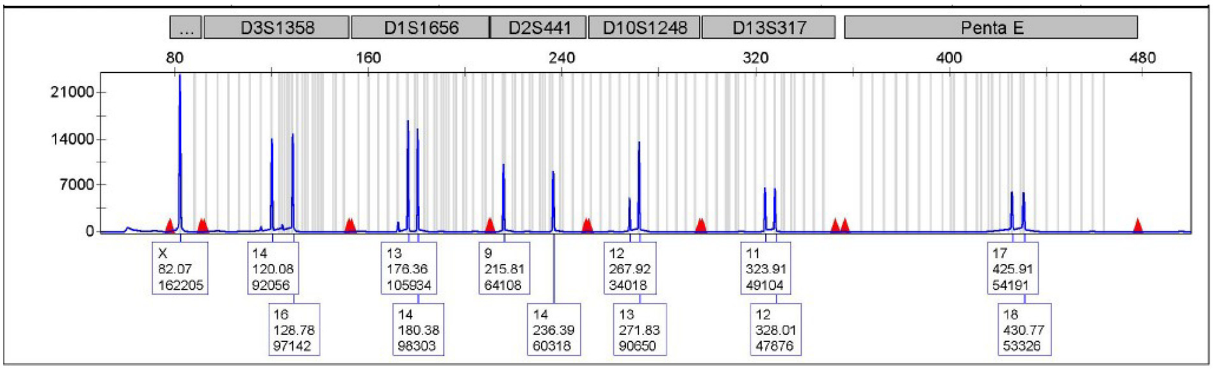

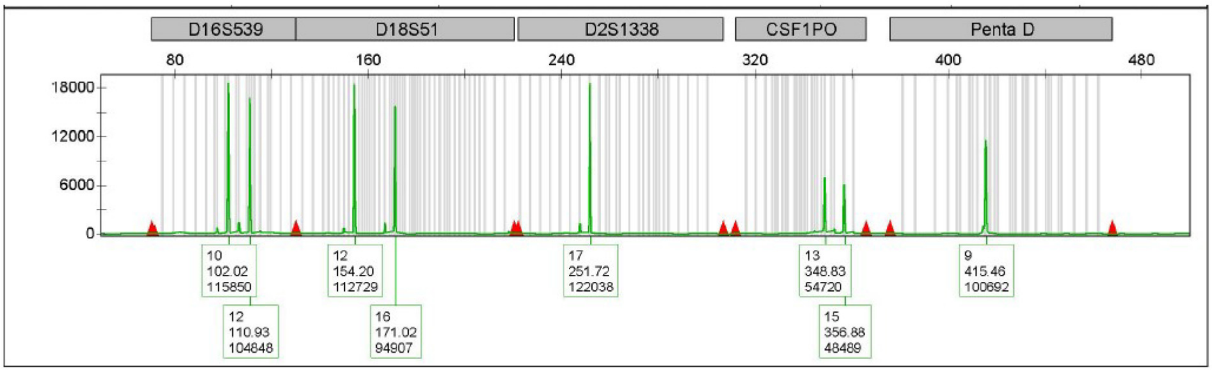

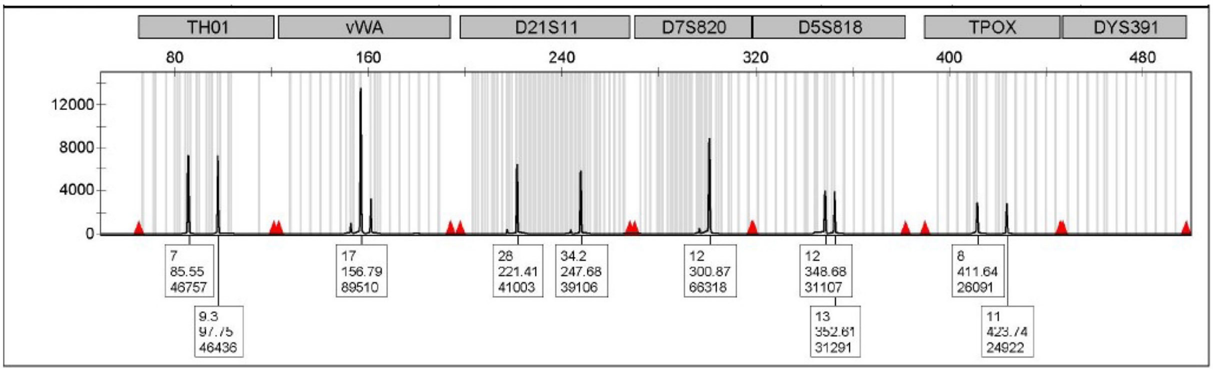

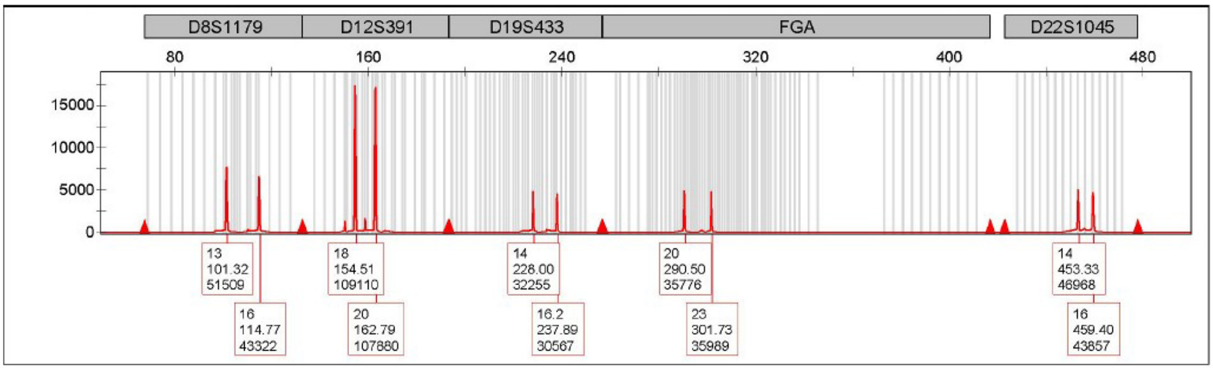

S2 Fig. DNA typing report of TOV-21G cells utilized in this study. Genomic DNA was extracted by Roche MagNA Pure Compact System and quantified by Nanodrop. Promega GenePrint 24 System was utilized to amplify DNA fragments of 24 unique STR loci. The sizes, heights, and plot areas of amplified DNA fragments were analyzed by ABI PRISM 3730 Genetic Analyzer and GeneMapper® Software V3.7.
