# Supplementary material for: Genetic impacts on thermostability of onco-lncRNA HOTAIR during the development and progression of endometriosis
Source: PLoS One. 2021 Mar 5;16(3):e0248168. doi: 10.1371/journal.pone.0248168 (PMC7935326; doi:10.1371/journal.pone.0248168)
Supplement: S3 Fig — (PDF) [file pone.0248168.s003.pdf]

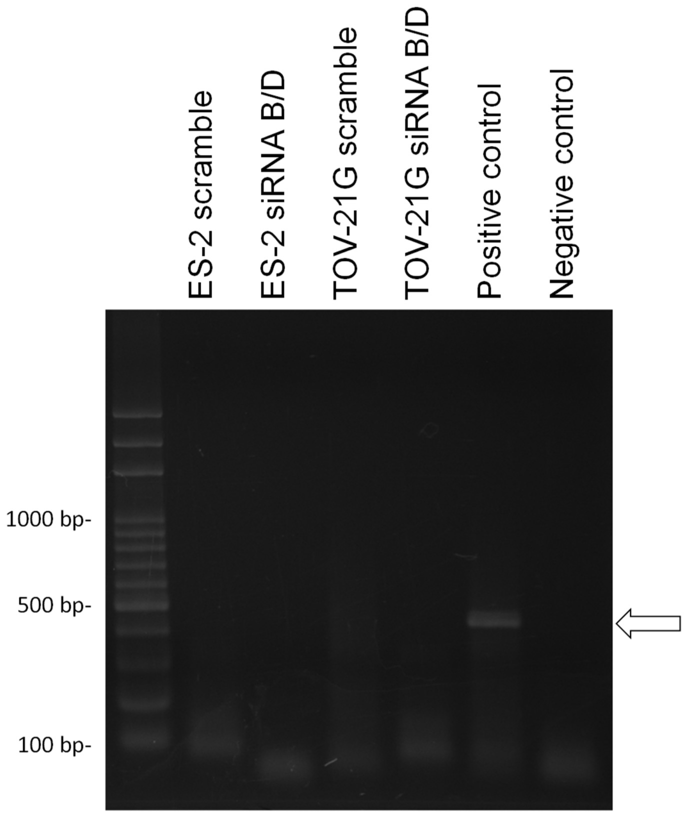

S3 Fig. PCR test for detecting mycoplasma contamination. A volume of 100 µl cell culture supernatant was taken from a dense culture (80-100% confluent) into a 1.5ml tube. Samples were denatured for 5 min at 95°C and the precipitates were spun down in a bench centrifuge at maximum speed for 2 min. PCR reaction was performed directly on the precipitates using the primers and amplification program shown in S3 Table. A positive control was previously prepared which had been identified as an infected culture, whereas fresh medium was utilized as a negative control. The arrow indicates the expected size (around 500 bp) of amplified DNA.
