# Supplementary material for: Genetic impacts on thermostability of onco-lncRNA HOTAIR during the development and progression of endometriosis
Source: PLoS One. 2021 Mar 5;16(3):e0248168. doi: 10.1371/journal.pone.0248168 (PMC7935326; doi:10.1371/journal.pone.0248168)
Supplement: S4 Fig — (PDF) [file pone.0248168.s004.pdf]

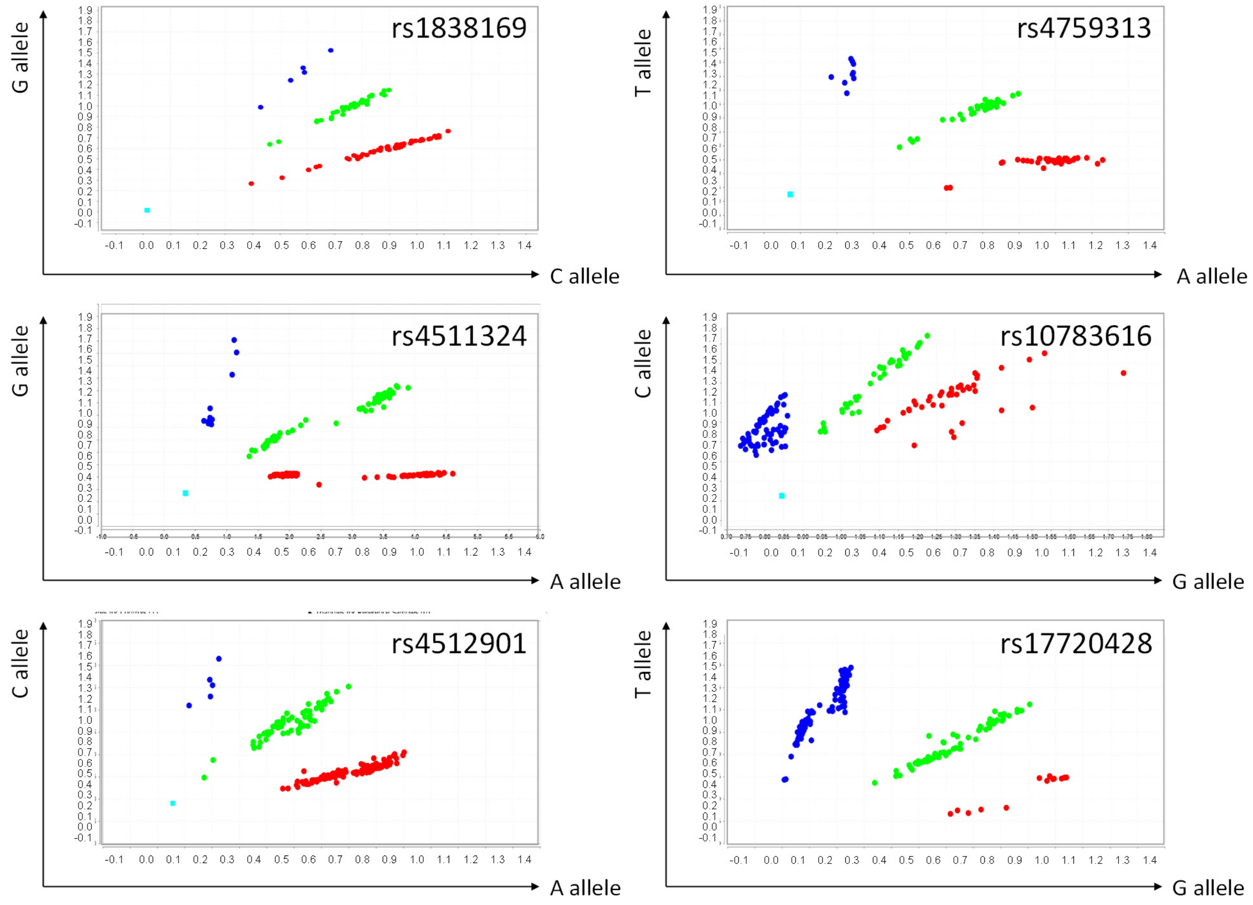

S4 Fig. Allelic discrimination plots of the six functional single-nucleotide polymorphisms (SNPs) in *HOTAIR* gene. DNA samples were prepared from PBMCs of patients and controls and genotyped by using *TaqMan* genotyping with probes listed in the S1 Table. The major (also the wild-type) and the minor alleles were detected either by 6-carboxyfluorescein (FAM)-labeled probes (blue) or by 2'-chloro-7'-phenyl-1,4-dichloro-6-carboxyfluorescein (VIC)-labeled probes (red). Samples with green dots indicate the heterozygous samples. "X" indicates the participant who failed to be genotyped.
