# Supplementary material for: Genetic impacts on thermostability of onco-lncRNA HOTAIR during the development and progression of endometriosis
Source: PLoS One. 2021 Mar 5;16(3):e0248168. doi: 10.1371/journal.pone.0248168 (PMC7935326; doi:10.1371/journal.pone.0248168)
Supplement: S5 Fig — (PDF) [file pone.0248168.s005.pdf]

rs1838169

Major allele (ΔG : -35.2)

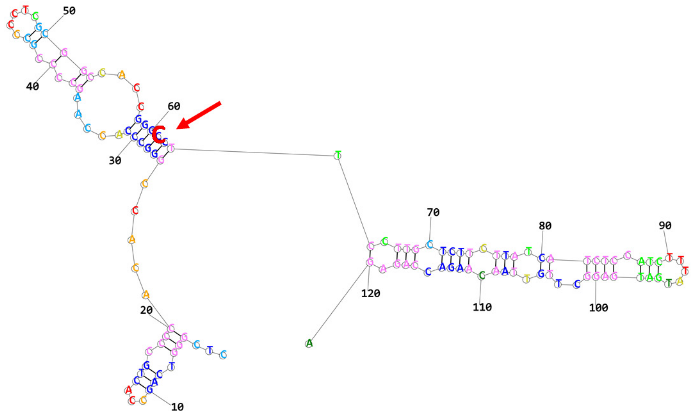

Minor allele (ΔG : -37.2)

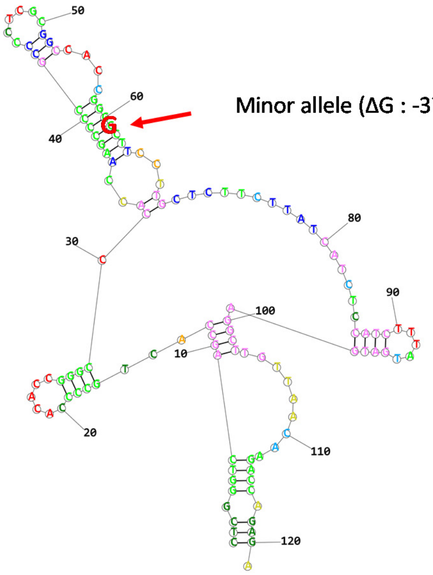

rs4511324

Major allele (ΔG : -34.1)

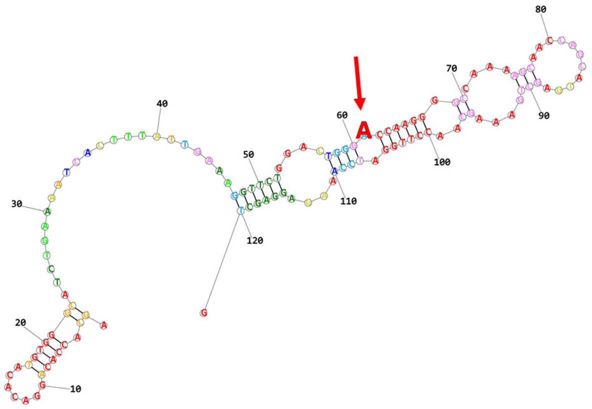

Minor allele (ΔG : -34.7)

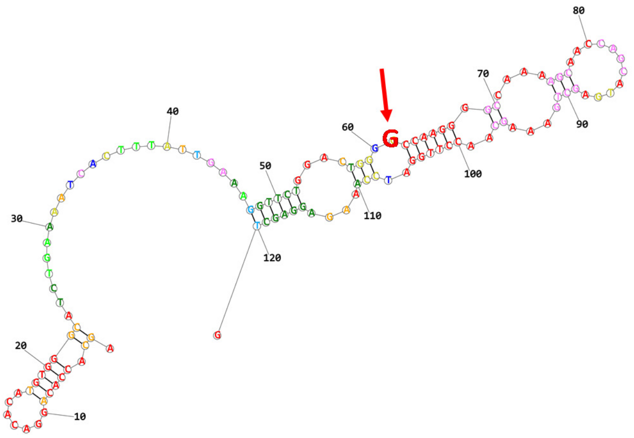

rs17720428

Major allele (ΔG : -24.9)

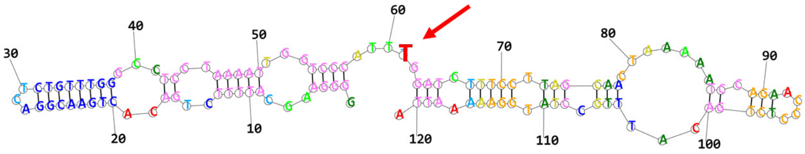

Minor allele (ΔG : -25.4)

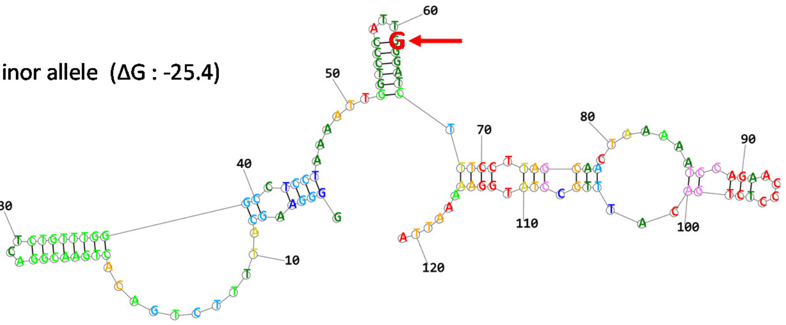

S5 Fig. Functional impacts of endometriosis-related risk alleles (SNPs at rs1838169, rs4511324 and rs17720428) on HOTAIR RNA structure. Partial RNA sequences with genetic substitutions at endometriosis-related risk SNPs (total 701 bp, the SNP site plus 350 bp upstream and 350 bp downstream sequences) were subjected to RNA stability/structure prediction by using MaxExpect (http://rna.urmc.rochester.edu/RNAstructureWeb/Servers/Predict1/Predict1.html) method[23]. Arrows indicate the reference major alleles and minor risk alleles for SNPs at rs1838169 (*upper* panel), rs4511324 (*middle*) and rs17720428 (*lower*).
