# Supplementary material for: Genetic impacts on thermostability of onco-lncRNA HOTAIR during the development and progression of endometriosis
Source: PLoS One. 2021 Mar 5;16(3):e0248168. doi: 10.1371/journal.pone.0248168 (PMC7935326; doi:10.1371/journal.pone.0248168)
Supplement: S6 Fig — (PDF) [file pone.0248168.s006.pdf]

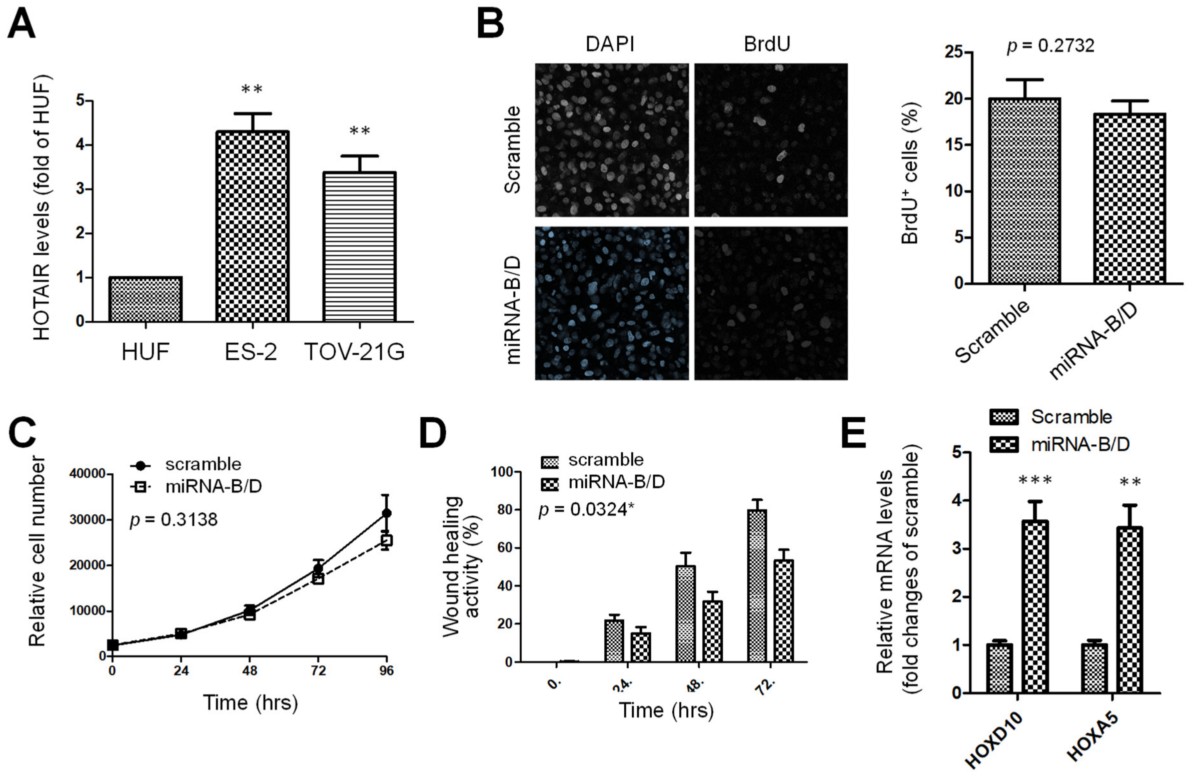

S6 Fig. Functional impacts of HOTAIR knockdown on survival activity of human uterine fibroblast (HUF) cells. (A) HOTAIR expression levels in the indicated cell lines were assayed by quantitative RT-PCR (S2 Table). Data were averages of triplicates and presented as means ± S.D. (B) BrdU uptake assay was performed in HUF cells transfected with scramble control or the mixture of anti-HOTAIR miRNA-B and miRNA-D (miRNA-B/D) (*left*). Positive cells were counted and averaged as means ± S.D. from 200 nuclei (*right*). (C) MTT assay and (D) wound healing assay were performed to investigate the impacts of HOTAIR knockdown by miRNA-B/D on cell growth and migration, respectively. (E) The expression levels of HOXD10 and HOXA5 were analyzed by QPCR in transfected cells 24 hrs after HOTAIR knockdown. Cells treated with scramble were utilized as the controls. Data were averages of triplicates and presented as means ± S.D. Data in (C) and (D) were averages of five replicates and presented as means ± S.D. For (A), (B) and (E), data from miRNA-B/D treated cells were compared to the ones from scramble control by using *t*-test. For (C) and (D) time course studies, two-way ANOVA was utilized to analyze statistical significance. The *p* values were presented as *: *p* value < 0.05, **: *p* value < 0.01, and ***: *p* value < 0.001.
