# Supplementary material for: Genetic impacts on thermostability of onco-lncRNA HOTAIR during the development and progression of endometriosis
Source: PLoS One. 2021 Mar 5;16(3):e0248168. doi: 10.1371/journal.pone.0248168 (PMC7935326; doi:10.1371/journal.pone.0248168)
Supplement: S1 Table — (PDF) [file pone.0248168.s007.pdf]

S1 Table. Summary of the functional SNPs in *HOTAIR*

SNP[a]	CHB MAF (%)	Major allele free energy (kcal/mol)[b]	Minor allele free energy (kcal/mol)[b]	ΔΔG (kcal/mol)	Location	ABI probe ID[c]
rs1838169	C: 84 G: 16	-35.2	-37.2	-2.0	exon/non-coding transcript variant	C___2104253_10
rs4511324	A: 76 G: 24	-34.1	-34.7	-0.6	intron/upstream transcriptional region	C__27952483_10
rs4512901	A: 85 C: 15	-23.9	-19.7	4.2	intron/upstream transcriptional region	C___2104249_10
rs4759313	A: 75 T: 25	-35.7	-34.2	1.5	intron/enhancer region	C__29123986_10
rs10783616	G: 65 C:35	-37.0	-32.8	4.2	intron/enhancer region	C___1124847_10
rs17720428	T: 85 G: 15	-24.9	-27.6	-2.7	exon/non-coding transcript variant	C___2104254_10
rs920778	A: 76 G:24	-14.8	-14.8	0	intron	F
rs7958904	G: 71 C: 29	-64.5	-64.5	0	exon	F
rs10783618	C: 76 T: 24	-29.0	-29.3	-0.3	intron	F

rs1899663	C: 85 A: 15	-33.8	-35.0	-1.2	intron	N/A
rs2366151	A: 76 G: 24	-23.7	-24.9	-1.2	downstream variant	N/A
rs2366152	A: 85 G: 15	-25.7	-25.0	0.7	exon	N/A
rs10783617	T: 75 G: 25	-24.5	-25.4	-0.9	intron	N/A
rs11170775	A: 75 G: 25	-32.0	-32.1	-0.1	intron	F
rs11170776	G: 85 T: 15	-26.3	-25.6	0.7	upstream variant	N/A
rs11613386	A: 86 G: 14	-37.9	-37.0	0.9	upstream variant	N/A
rs12312094	C: 84 G: 16	-23.8	-25.0	-1.2	exon	N/A
rs17105613	T: 85 C: 15	-33.4	-34.4	-1.0	intron	N/A
rs200349340	G: 77 T: 23	-22.2	-21.7	0.5	exon	N/A

[a]SNPs in *HOTAIR* with a minor allele frequency (MAF) more than 10% in Beijing Chinese Han (CBH) population

[b]The predicted RNA structures and the estimated free energy were generated by MaxExpect method (http://rna.urmc.rochester.edu/RNAstructureWeb/Servers/Predict1/Predict1.html)

[c]SNPs with ABI probe IDs were selected for this study. F: failed to meet the criteria ($\Delta\Delta G > 0.5$ or < -0.5); N/A: no *Taqman* probe available
