# Supplementary material for: Genetic impacts on thermostability of onco-lncRNA HOTAIR during the development and progression of endometriosis
Source: PLoS One. 2021 Mar 5;16(3):e0248168. doi: 10.1371/journal.pone.0248168 (PMC7935326; doi:10.1371/journal.pone.0248168)
Supplement: S2 Table — (PDF) [file pone.0248168.s008.pdf]

S2 Table. Primer sequences and amplification program for quantitative RT-PCR reactions in this study.

Gene name	Reference sequence	Forward primer sequence[a,b]	Reverse primer sequence[a,b]
HOTAIR	NR_047517.1	5'- AATAGACATAGGAGAACACTT -3'	5'- AATCTTAATAGCAGGAGGAA -3'
HOXD10	NM_002148.4	5'- ATATACCTCAAGTAGACA -3'	5'- GATTCTTCCTTAATGTTG -3'
HOXA5	NM_019102.4	5'- AAGTGTTCCTGTCTCAATAGC -3'	5'- TGTCTCATCAAGTCACCTCTA -3'
GAPDH	NM_001289745	5'-CTCTGGTAAAGTGGATATTGT-3'	5'-GGTGGAATCATATTGGAACA-3'
ex-HOTAIR[c]	HOTAIR vectors	5'-AAGAACGCAATTTCAATGT-3'	5'-CCGAATTAATACGACCACTA-3'
puromycin	HOTAIR vectors	5'-GCTCGTAGAAGGGGAGGTTG-3'	5'-ACAGATGGAAGGCCTCCTG-3'

[a]The SYBR Green-based primers were designed by OligoArchitect[TM] online (http://www.oligoarchitect.com/SYBRGreenSearchServlet).

[b]QPCR cycles were performed by using an ABI 2720 Thermal Cycler (Applied Biosystems, Carlsband, CA) and the cycling conditions were: 95°C for 10 mins, followed by 40 cycles of 95°C for 15 seconds, 55°C for 15 seconds, 72°C for 15 seconds, and finally 72°C for 5 mins.

[c]The PCR reaction for ex-HOTAIR only detects exogenous HOTAIR expressed by the vector, but not endogenous HOTAIR because the primer set recognizes the joint region between 3'-end of HOTAIR and the vector backbone.
