# Supplementary material for: Genetic impacts on thermostability of onco-lncRNA HOTAIR during the development and progression of endometriosis
Source: PLoS One. 2021 Mar 5;16(3):e0248168. doi: 10.1371/journal.pone.0248168 (PMC7935326; doi:10.1371/journal.pone.0248168)
Supplement: S3 Table — (PDF) [file pone.0248168.s009.pdf]

S3 Table. Primer sequences and PCR amplification program for site-directed mutagenesis in this study[a,b,c]

SNP site	Forward primer sequence (5' to 3')	Reverse primer sequence (5' to 3')
**Step-1 PCR to generate fragments A and B**		
rs1838169-A	CGCC*CACGTG*AAGGCTGCCGAC	ATAAGAAGAGCAAGGAAG**G**CCCGGTGGCCGCGAGGGG
rs1838169-B	CCCCTCGCGGCCACCGGG**C**CTTCCTTGCTCTTCTTAT	GTGTAACAG*GCAGGTG*GATTCCTG
rs17720428-A	CGCC*CACGTG*AAGGCTGCCGAC	CCTAAAATTGGTCCCA**G**TTGGATCTTTCCTTAGCAAC
rs17720428-B	GTTGCTAAGGAAAGATCC**C**AATGGGACCAATTTTAGG	GTGTAACAG*GCAGGTG*GATTCCTG
**Step-2 overlap extension PCR to join fragments A and B together**		
rs1838169 & rs17720428	CGCC*CACGTG*AAGGCTGCCGAC Pml1 site	GTGTAACAG*GCAGGTG*GATTCCTG Aar1 site

[a]The bold letters with underlines in primers indicate the substitutive residues whereas the Italic letters with underlines indicate the restriction enzyme sites.

[b]For the Step-1 PCR, 0.1 ng wild type plasmid was utilized as the template for PCR. For the Step-2 overlap extension PCR, 10 ng fragments A and B were used as the template.

[b]PCR cycles were performed by using an ABI 2720 Thermal Cycler (Applied Biosystems, Carlsband, CA) and the cycling conditions were: 95°C for 10 mins, followed by 30 cycles of 95°C for 15 seconds, 55°C for 15 seconds, 72°C for 1 min, and finally 72°C for 5 mins.
