# Supplementary material for: Genetic impacts on thermostability of onco-lncRNA HOTAIR during the development and progression of endometriosis
Source: PLoS One. 2021 Mar 5;16(3):e0248168. doi: 10.1371/journal.pone.0248168 (PMC7935326; doi:10.1371/journal.pone.0248168)
Supplement: S4 Table — (PDF) [file pone.0248168.s010.pdf]

S4 Table. Primer sequences and amplification program for PCR-based mycoplasma test.

**Primer**	**Primer sequence[26]**
Forward primer: mixtures of the following 6 primers	
Myco-F1	CGCCTGAGTAGTACGTTCGC
Myco-F2	CGCCTGAGTAGTACGTACGC
Myco-F3	TGCCTGAGTAGTACATTCGC
Myco-F4	TGCCTGGGTAGTACATTCGC
Myco-F5	CGCCTGGGTAGTACATTCGC
Myco-F6	CGCCTGAGTAGTATGCTCGC
Reverse primer: mixtures of the following 3 primers	
Myco-R1	GCGGTGTGTACAAGACCCGA
Myco-R2	GCGGTGTGTACAAAACCCGA
Myco-R3	GCGGTGTGTACAAACCCCGA

The PCR program: initial denaturation at 95°C for 2 min; five cycles of 94°C-50°C-72°C, each step for 30 sec; thirty cycles of 94°C-56°C-72°C, each step for 30 sec.
