# Supplementary material for: Genetic impacts on thermostability of onco-lncRNA HOTAIR during the development and progression of endometriosis
Source: PLoS One. 2021 Mar 5;16(3):e0248168. doi: 10.1371/journal.pone.0248168 (PMC7935326; doi:10.1371/journal.pone.0248168)
Supplement: S5 Table — (PDF) [file pone.0248168.s011.pdf]

S5 Table. Target sequences of siRNA vectors used in this study.

siRNA vector[a]	Target sequence
scramble	5’-GGGTGAACTCACGTCAGAA-3’
siRNA-A	780-GAGGAAAAGGGAAAATCTA
siRNA-B	801-GAACGGGAGTACAGAGAGA
siRNA-C	840-CCACATGAACGCCCAGAGA
siRNA-D	1005-TAACAAGACCAGAGAGCTG

[a]Vector set of siRNAs (siRNA-A to siRNA-D) were designed to knockdown HOTAIR (NR_003716) expression; scramble vector was used as the control.
